# Phylogenetic analysis of three genes of Penguinpox virus corresponding to Vaccinia virus G8R (VLTF-1), A3L (P4b) and H3L reveals that it is most closely related to Turkeypox virus, Ostrichpox virus and Pigeonpox virus

**DOI:** 10.1186/1743-422X-6-52

**Published:** 2009-05-08

**Authors:** Olivia Carulei, Nicola Douglass, Anna-Lise Williamson

**Affiliations:** 1 Institute of Infectious Disease and Molecular Medicine and Department of Clinical Laboratory Sciences, Faculty of Health Sciences, University of Cape Town, Cape Town 7925, South Africa; 2 National Health Laboratory Service, Groote Schuur Hospital, Observatory, Cape Town 7925, South Africa

## Abstract

Phylogenetic analysis of three genes of Penguinpox virus, a novel Avipoxvirus isolated from African penguins, reveals its relationship to other poxviruses. The genes corresponding to Vaccinia virus G8R (VLTF-1), A3L (P4b) and H3L were sequenced and phylogenetic trees (Neighbour-Joining and UPGMA) constructed from MUSCLE nucleotide and amino acid alignments of the equivalent sequences from several different poxviruses. Based on this analysis, PEPV was confirmed to belong to the genus Avipoxvirus, specifically, clade A, subclade A2 and to be most closely related to Turkeypox virus (TKPV), Ostrichpox virus (OSPV)and Pigeonpox virus (PGPV).

## Background

Interest in the avipoxviruses, notably *Fowlpox virus *(FWPV) and *Canarypox virus *(CNPV) has increased due to their successful use as vaccines on commercial flocks and their extensive use and testing as vaccine vectors [1-8]. The genomes of both FWPV and CNPV have been sequenced and comparison reveals a high level of divergence with significant differences between orthologous ORFs and the terminal, variable genomic regions [9,10]. Analysis of the thymidine kinase gene showed only 64% amino acid identity between FWPV and CNPV compared to 97% amino acid identity amongst the orthopoxviruses and 84% within the *Leporipoxvirus *genus [11]. This level of divergence is commonly seen between different *Chordopoxvirus *genera suggesting that the species within the *Avipoxvirus *genus are highly divergent. A novel avipoxvirus, Penguinpox virus (PEPV) was isolated from an African penguin (*Spheniscus demersus*) that was brought into the Southern African Foundation for the Conservation of Coastal Birds (SANCCOB) [12]. Lesions around the eyes, typical of avipoxvirus infection were noted and scrapings were taken. Virus was cultured from these scrapings and histological studies and restriction enzyme profile comparison to other known avipoxviruses, namely FWPV, CNPV, *Turkeypox virus *(TKPV) and Quailpox virus, confirmed that it was indeed a novel avipoxvirus [12]. Infectivity studies of different mammalian cell lines (CV-1, Vero, MDBK, RK-13, HeLa and HEF) and chick embryo fibroblasts (CEFs) showed that early stages of virus replication were supported, but no infectious progeny virus could be recovered [13]. It is currently unclear as to why PEPV cannot be successfully passaged in CEFs as CEFs have been shown to support replication of both FWPV and CNPV viruses. Also reported was the fact that PEPV transcriptases could recognize the *Vaccinia virus *(VACV) derived late promoter P11 linked to the β-galactosidase reporter gene, resulting in transient gene expression.

## Results and discussion

One highly conserved gene, VLTF-1 (VACV G8R; *fpv*126 locus), was chosen for analysis in order to position PEPV in the larger chordopoxvirus group. Two additional genes, which are less highly conserved (P4b (VACV A3L; *fpv*167 locus) and the virion envelope protein p35 (VACV H3L; *fpv*140 locus)) were selected for analysis in order to determine the relationship of PEPV to other avipoxviruses previously analysed at these loci [14]. The analysis involved MUSCLE [15] amino acid and nucleotide alignments and construction of UPGMA and Neighbour-Joining [16] phylogenetic trees based on these alignments.

**Figure 1:**
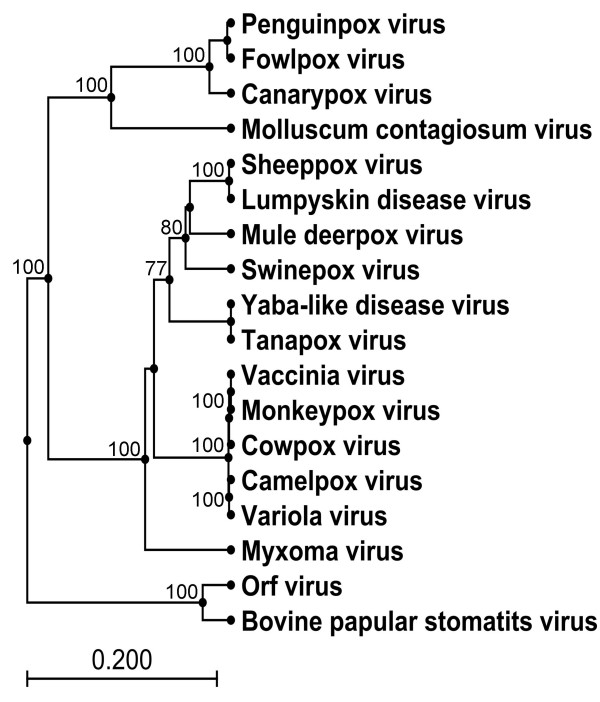
**Phylogenetic tree based on alignment of VLTF-1 (VACV G8R; *fpv*126 locus) amino acid sequences**. Neighbour-Joining phylogenetic tree constructed from the MUSCLE alignment of the amino acid sequences of the VLTF-1 gene (*fpv*126 locus) from 18 poxviruses. (Bootstrap values from 1000 replicate samplings are shown).

The VLTF-1 gene encodes a late transcription factor, which is highly conserved amongst all poxviruses and is the most conserved protein between FWPV and CNPV with 95% amino acid identity [10]. The nucleotide and amino acid sequences of 18 poxviruses representing all eight *Chordopoxvirus *genera were analysed at this locus. The overall tree topologies are as previously reported [17] and this analysis shows PEPV to belong to the *Avipoxvirus *genus, grouping with FWPV, in a separate clade from CNPV, with strong bootstrap support in both UPGMA and N-J trees (N-J tree shown below in Figure 1). PEPV showed 96% amino acid identity to FWPV and 92% identity to CNPV. The nucleotide identity was lower with 92% identity to FWPV and 84% identity to CNPV. Divergence is therefore more easily detected in the nucleotide sequences due to the increased number of changes and nucleotide sequences were therefore used for analysis of the P4b and envelope protein, p35 genes.

**Figure 2:**
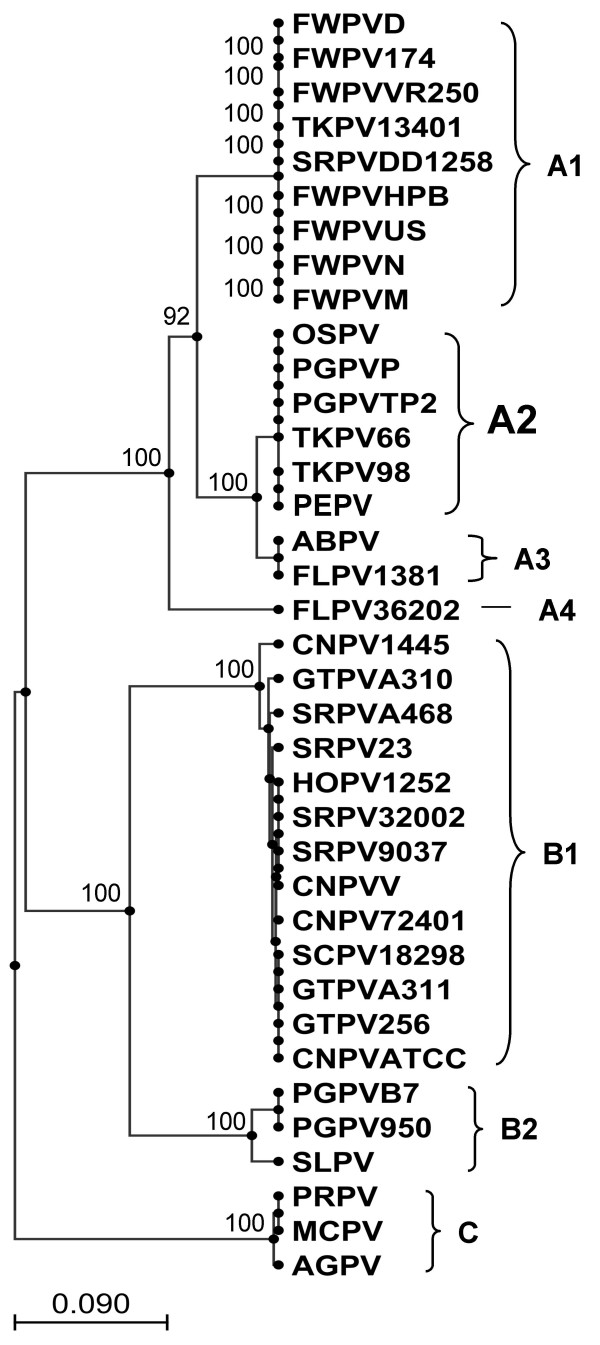
**Phylogenetic tree based on alignment of P4b (VACV A3L; *fpv*167 locus) DNA sequences**. Neighbour-Joining phylogenetic tree constructed from the MUSCLE alignment of the nucleotide sequences of the P4b gene (*fpv*167 locus) of 37 Avipoxvirus isolates from 17 species of bird. (Bootstrap values from 1000 replicate samplings are shown). (ABPV = Albatrosspox virus; AGPV = Agapornispox virus; CNPV = Canarypox virus; FLPV = Falconpox virus; FWPV = Fowlpox virus; GTPV = Great titpox virus; HOPV = Houbara bustardpox virus; MCPV = Macawpox virus; OSPV = Ostrichpox virus; PEPV = Penguinpox virus; PGPV = Pigeonpox virus; PRPV = Parrotpox virus; SCPV = Stone curlewpox virus; SLPV = Starlingpox virus; SRPV = Sparrowpox virus; TKPV = Turkeypox virus).

The P4b gene encodes a 75.2 kDa virion core protein, which is highly conserved amongst all poxviruses [18] and this locus has been used previously in phylogenetic studies of avipoxviruses [14,19-21] to differentiate between major clades A, B and C as well as minor clades A1, A2, A3, A4, B1 and B2. Figure 2 shows an N-J tree based on a MUSCLE nucleotide alignment of partial sequences (truncated to 450 nt to ensure that all sequences were of equal length) of P4b orthologues from 37 avipoxvirus isolates from 17 different species of bird. This tree gives better resolution of the *Avipoxvirus *genus and shows PEPV to belong to the FWPV-like group of viruses (clade A) as opposed to the CNPV-like group of viruses (clade B) or the Psittacine viruses (clade C) in both N-J and UPGMA trees. FWPV and CNPV orthologues showed an average of 75% nucleotide identity to each other for the various isolates at this locus. PEPV showed an average of 90% nucleotide identity to the various FWPV isolates and 74% identity to the various CNPV isolates. PEPV was found to belong to subclade A2 with 98% similarity to Falconpox virus (FLPV) and Albatrosspox virus (ABPV), and 100% homology to both the TKPV isolates (TKPV66 and TKPV98), the Ostrichpox virus (OSPV) isolate and two of the *Pigeonpox virus *(PGPV) isolates (PGPVTP2 and PGPVP).

**Figure 3:**
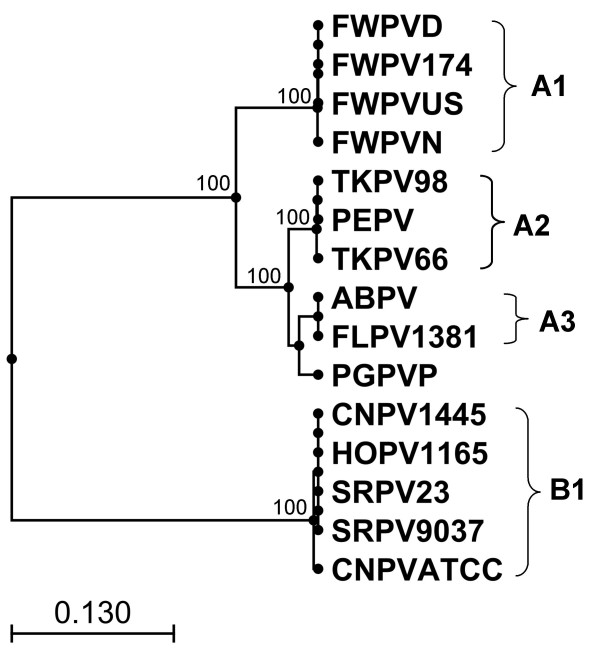
**Phylogenetic tree based on alignment of H3L (*fpv*140 locus) DNA sequences**. Neighbour-Joining phylogenetic tree constructed from the MUSCLE alignment of the nucleotide sequences of the H3L gene (*fpv*140 locus) from 15 Avipoxvirus isolates from 9 species of bird. (Bootstrap values from 1000 replicate samplings are shown). (ABPV = Albatrosspox virus; CNPV = Canarypox virus; FLPV = Falconpox virus; FWPV = Fowlpox virus; HOPV = Houbara bustardpox virus; PEPV = Penguinpox virus; PGPV = Pigeonpox virus; SRP = Sparrowpox virus; TKPV = Turkeypox virus).

Figure 3 shows an N-J tree based on the MUSCLE nucleotide alignment of orthologues of the envelope protein p35, which is an immunodominant antigen. PEPV was found to cluster with the TKPV isolates with strong bootstrap support while the pigeon isolate PGPVP clustered with the isolates from albatross and falcon in both N-J and UPGMA trees. PEPV was found to have 99% nucleotide identity with both TKPV isolates in subclade A2, compared to 95% identity with ABPV and FLPV and 94% with PGPVP.

Taken together these analyses confirm that PEPV belongs to the genus *Avipoxvirus*, and go on to show that PEPV belongs to subclade A2 with the highest degree of similarity to TKPV isolates 98 and 66. Because there was no OSPV sequence available for the envelope protein p35 we can only speculate that PEPV and OSPV are highly similar based on the degree of similarity at the P4b gene. Previous work has shown that OSPV, TKPV and FWPV are genetically, antigenically and biologically related. Ostrich chicks housed in an enclosure that had previously housed pox infected turkeys became infected with a poxvirus and immunisation of susceptible chickens with this poxvirus protected them from FWPV challenge [22]. OSPV isolated from ostriches in Israel was found to produce productive infection in turkeys and furthermore, ostrich chicks in both Israel and South Africa could be protected by immunisation with FWPV vaccine [23,24]. The host range of TKPV has been reported to include both ostrich and Humboldt penguin (a close relative of the African penguin), though this is unconfirmed [25]. Analysis of a PEPV IL-10 like protein (data not shown) shows this protein to be most closely related to turkey (*Meleagris gallopavo*) and chicken (*Gallus gallus*) IL-10, with 27% amino acid identity followed by the IL-10 like protein found in CNPV with 26% amino acid identity. The CNPV IL-10-like protein showed only 20% identity to the proteins from chicken and turkey. PEPV infection is evident in juvenile African penguins especially in the summer months when the penguins spend the majority of their time on land (moulting and/or nesting) and there are large numbers of mosquitoes present, which act as mechanical vectors to transmit virus (personal communication Dr. Nola Parsons, SANCCOB). Further research is underway to determine whether outbreaks in different avian species are caused by the same virus or by different but closely related viral species.

## Materials and methods

PEPV was grown on the chorioallantoic membranes of embryonated hens' eggs as described previously [13] to produce a viral stock from which DNA was extracted. Viral DNA was extracted by conventional methods as described previously [12] with the following modifications to the lysis buffer: 10% N-lauryl sarcosinate, 50 mM Tris pH7.8, 200 mM β-mercaptoethanol and no SDS. The PEPV genomic DNA was sequenced using the Roche/454 GS-FLX system and all bioinformatics analysis was performed using the CLC Bio Main Workbench.

Accession numbers of all loci used in this study (Table 1).

**Table 1:** Accession numbers of all loci used in this study

**Isolate**	**G8R (VLTF-1)**	**A3L (P4b)**	**H3L**
ABPV	-----	AM050392	AM071388
AGPV	-----	AY530311	-----
BPSV	AY386265	-----	-----
CMPV	AF438165	-----	-----
CNPVATCC	AY318871	AY318871	AY318871
CNPV72401	-----	AY530306	-----
CNPV1445	-----	AM050375	AM071512
CNPVV	-----	AM050384	-----
CPXV	AF482758	-----	-----
FLPV36202	-----	AY530306	-----
FLPV1381	-----	AM050376	AM071515
FWPVVR250	-----	AY453172	-----
FWPVUS	AF198100	AF198100	-----
FWPVHPB	-----	AY530302	-----
FWPVFP9	-----	AJ581527	AJ581527
FWPV174	-----	AM050377	AM071393
FWPVD	-----	AM050380	AM071395
FWPVN	-----	AM050379	AM071394
FWPVM	-----	AM050378	-----
GTPV256	-----	AY453175	-----
GTPVA310	-----	AY453173	-----
GTPVA311	-----	AY453174	-----
HOPV1252	-----	AM050381	-----
HOPV1165	-----	-----	AM071513
LSDV	AF325528	-----	-----
MCPV		AM050382	-----
MCV	U60315	-----	-----
MDPV	AY689436	-----	-----
MPXV	AF380138	-----	-----
MYXV	AF170726	-----	-----
ORFV	AY386264	-----	-----
OSPV	-----	AY530305	-----
PEPV	FJ948104	FJ948105	FJ948106
PGPVB7	-----	AY453177	-----
PGPVTP2	-----	AY530303	-----
PGPVP	-----	AM050385	AM071389
PGPV950	-----	AM050386	-----
PRPV	-----	AM050383	-----
SCPV18298	-----	AY530310	-----
SLPV	-----	AM050391	-----
SPPX	AY077832		-----
SRPVDD1258	-----	AY530307	-----
SRPV32002	-----	AY530308	-----
SRPVA468	-----	AY453176	-----
SRPV9037	-----	AM050389	AM071511
SRPV23	-----	AM050390	AM071510
SWPV	AF410153	-----	-----
TANV	EF420156	-----	-----
TKPV13401	-----	AY530304	-----
TKPV66	-----	AM050387	AM071390
TKPV98	-----	AM050388	AM071391
VACV	AY243312	-----	-----
VARV	X69198	-----	-----
YLDV	AJ293568	-----	-----

For further information on sequences used in this analysis please refer to [14].

## Competing interests

The authors declare that they have no competing interests.

## Authors' contributions

OC carried out the molecular genetic, sequence alignment and phylogenetic studies and drafted the manuscript. ND and ALW participated in the design of the study, analyses and interpretation of data and revision of the manuscript. All authors read and approved the final manuscript.
